# Anatomic tunnel placement can be achieved with a modification to transtibial technique in single bundle anterior cruciate ligament reconstruction: A cadaver study

**DOI:** 10.1371/journal.pone.0180860

**Published:** 2017-07-31

**Authors:** Joon Kyu Lee, Sahnghoon Lee, Ki Tae Kim, Myung Chul Lee

**Affiliations:** 1 Department of Orthopaedic Surgery, Hallym University Sacred Heart Hospital, Anyang, Korea; 2 Department of Orthopaedic Surgery, Seoul National University Hospital, Seoul, Korea; Mayo Clinic Minnesota, UNITED STATES

## Abstract

Placing the tunnels in the anatomic positions is important for successful restoration of knee function after anterior cruciate ligament reconstruction (ACLR). It has been shown that it is difficult to place the tunnels in the anatomic position using the transtibial technique. The purpose of this study was to evaluate the effect of each step of our modified transtibial technique (mTT) on the positioning of the femoral tunnel so as to assess whether the mTT could achieve anatomic placements of the tunnels without tibial tunnel expansion. Ten fresh-frozen cadaveric knees were used. First, the tibial tunnel was created in the center of ACL footprint. Then, a pin was inserted through the tibial tunnel using a femoral guide by four stepwise techniques: transtibial technique, additional anterior drawer force applied to the proximal tibia, another additional varus force applied to the tibia and finally, additional external rotation of the tibia and the femoral guide (mTT). Then, tibial tunnel was re-reamed using mTT with 10mm-diameter reamer. The pin positions in each technique on the femur were evaluated by the quadrant method and shapes of the tibial tunnel apertures were evaluated. Femoral pin positions in the four techniques were 23.6±4.5%, 28.4±3.4%, 30.1±3.8%, 33.2±4.5% in the superior-inferior position, and 23.9±4.3%, 26.2±3.7%, 32.0±4.3%, 36.9±4.8% in the anterior-posterior position, respectively. Pin position shifted to more inferior and posterior position with each step of mTT (all p values comparing superior-inferior and anterior-posterior positions of each step with positions of previous step were 0.008 or less). Using mTT, tibial tunnel aperture was 10.5±0.3mm wide and 12.9±1.1mm long. In conclusion, anatomic placements of femoral tunnels in ACLR without excessive tibial tunnel expansion could be achieved using the mTT.

## Introduction

There has been increased focus on the anatomic positioning of the tunnels in anterior cruciate ligament (ACL) reconstruction [1–6]. Although the transtibial technique is a generally accepted technique for creating tunnels, there have been concerns regarding the feasibility of placement of tunnels in the anatomic position using this technique [6–8]. There also were concerns that posterior wall blowout might occur with the transtibial technique when the femoral tunnel was targeted anatomically [9]. Due to the restriction imposed by the tibial tunnel, the femoral tunnel tends to be placed at a more anterior and superior position than that in the native ACL insertion site [10, 11]. Therefore, it seems quite clear that the modification to the transtibial technique should result in movement of the femoral tunnel to a more posterior and inferior position. To verify the exact positions on the femur and tibia where the tunnel should made, there have been several cadaver studies that reported the anatomic positions of the native ACL footprint [12–21]. The data reported in these studies should be considered as references when evaluating the tunnel positions by a certain technique to be anatomic or not.

To achieve anatomic ACL reconstruction, several modifications to the transtibial technique were suggested in order to place the femoral tunnel at a more posterior and inferior position. Some of the early efforts were focused mainly on the tibial tunnel starting point and obliquity of the tunnel [22–25]. Recently, there was a report which made modification to transtibial technique by maneuvering tibia during femoral tunnel creation. Their result was encouraging that they could place femoral tunnel at a point similar to anteromedial portal technique which were known to be favorable to place femoral tunnel at an anatomic position [26]. We also modified the transtibial technique and have used this modified transtibial technique (mTT) in practice [27]. There are three steps involved in the modification of the transtibial technique in order to move the femoral tunnels to a more posterior and inferior position; First, applying an anterior drawer force to the proximal tibia, second, applying an additional varus force to the proximal tibia and third, applying another additional external rotation force to the proximal tibia and externally rotating the femoral offset guide while inserting the femoral guide pin. The position of the tibial tunnel articular exit point was aimed at the center of ACL anteromedial and posterolateral bundle footprints.

The purpose of this study was to evaluate the effect of each step of our mTT on the positioning of the femoral tunnel in single bundle ACL reconstruction to elucidate whether anatomic positioning of the tunnel was possible using the mTT. We also evaluated tibial tunnel position, which should be in anatomic position for mTT to be meaningful, and the surgically created tibial tunnel aperture shape to verify the tibial tunnel expansion.

## Materials and methods

The femoral and tibial tunnel positions and the shape of tibial tunnel aperture by the mTT were studied in a step by step manner in 10 fresh-frozen human cadaveric knees. The cadaveric knees were from five donors (1 male, 4 females) and the average age of the donors was 77.6 years ranging from 67 years to 85 years. The average height of the donors was 154.6cm ranging from 147cm to 164cm. The knees with a history of previous surgery that could have affected the status of the ligament of the knee joint were excluded from the analysis.

**Fig 1:**
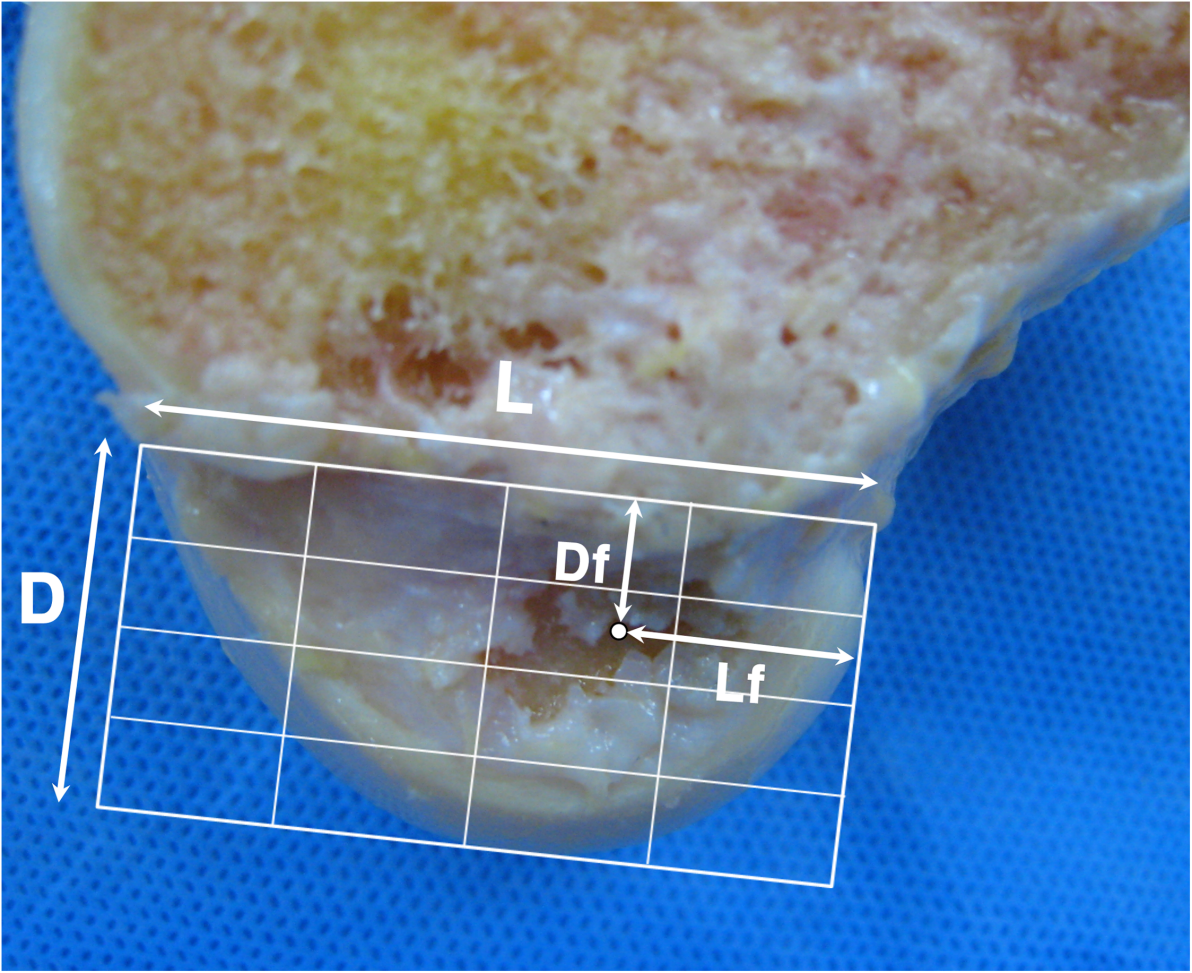
Quadrant method used to evaluate the femoral tunnel guide pin position. The position is defined as the ratio Df/D and Lf/L (D: total depth of the intercondylar notch; Df: distance from the pin position to the intercondylar notch; L: total length of the lateral condyle; Lf: distance from the pin position to the most superior contour of the lateral condyle).

**Fig 2:**
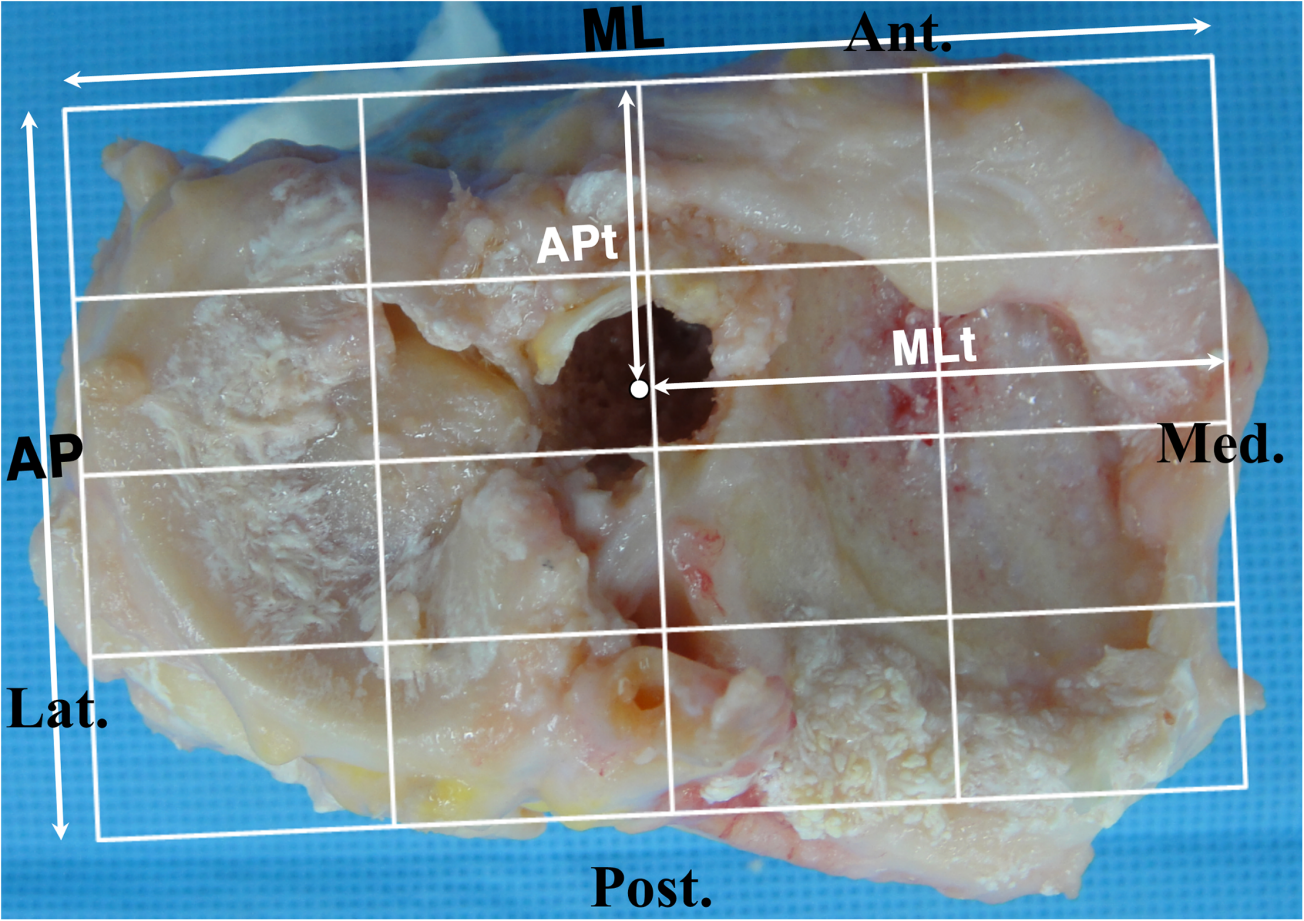
Quadrant method used to evaluate the center of tibial tunnel articular aperture. The position is defined as the ratio MLt/ML and APt/AP (ML: mediolateral width of the tibial plateau surface; MLt: distance from the center of the tibial tunnel aperture to the most medial contour of the tibial plateau surface; AP: anteroposterior length of the tibial plateau surface; APt: distance from the center of the tibial tunnel aperture to the most anterior contour of the tibial plateau surface).

**Fig 3:**
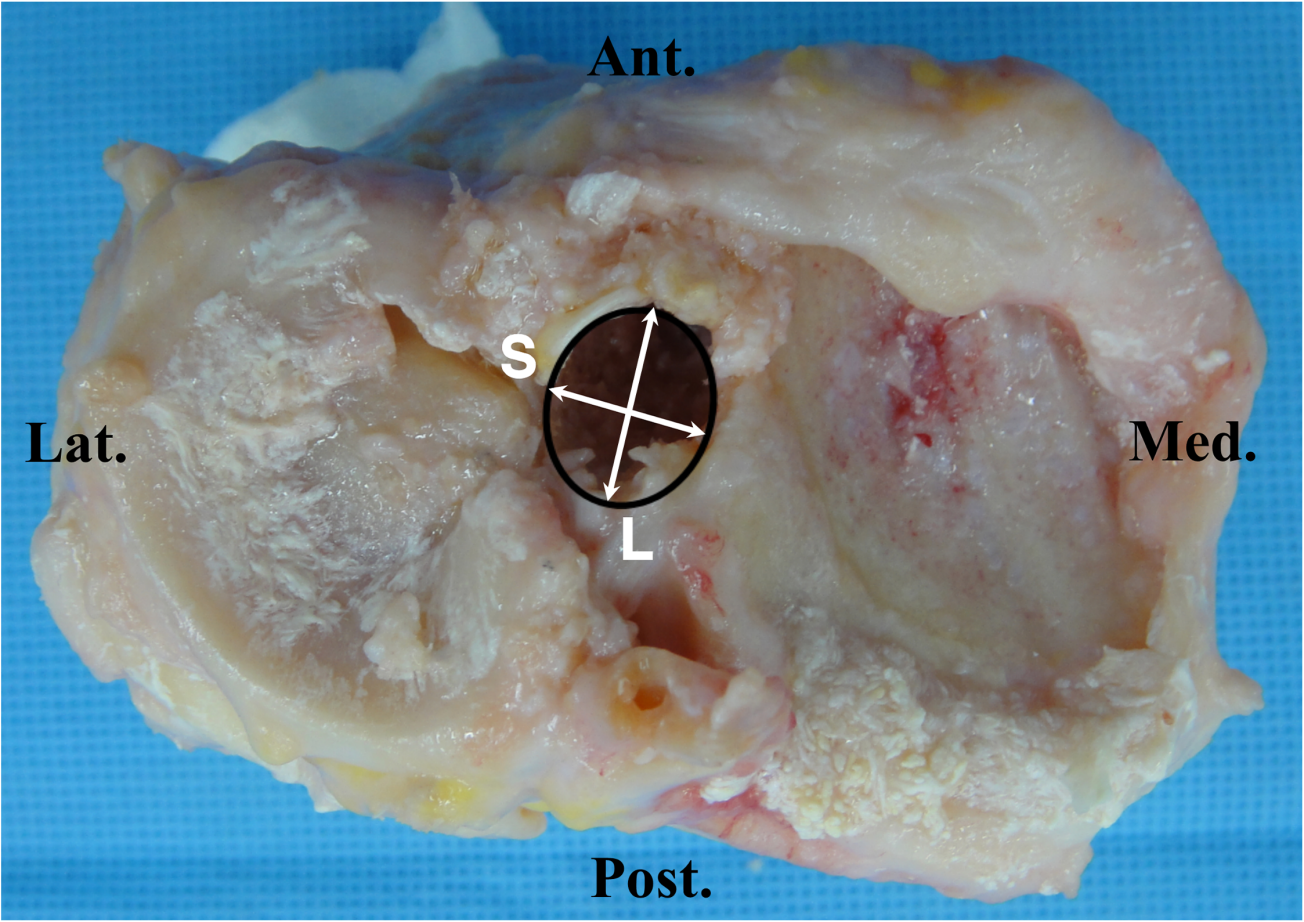
Shapes of the tibial tunnels created by the modified transtibial technique. Lengths of the long axis (L) and short axis (S) were measured.

All major procedures were performed by a single surgeon (*) with the help of two assistants. Identical techniques were used for each specimen. A medial parapatellar arthrotomy was used to expose the knee joint. The patella was everted and the ACL was excised completely. The other soft tissue structures were preserved as much as possible. The tibial tunnel was created first. The tibial tunnel entry point was set at 4~5cm distal to the joint line, 2~3cm posteromedial to the tibial tuberosity, 1cm superior to the attachment site of pes anserinus and just anterior to the medial collateral ligament (MCL). A guide pin was inserted at an angle of 60° to the tibial plateau using a tibial drill guide (Acufex, Andover, MA) aimed at the center of ACL anteromedial and posterolateral bundle footprints. Because the knee joint was exposed, tibial tunnel placements were done accurately and easily at the anatomic position. A 10mm tibial tunnel was reamed. Then, the patella was reduced and a pin was inserted through the tibial tunnel using a femoral guide with an off-set length of 7mm(Acufex) in a step by step manner by using four techniques. Regarding the knee positions during the maneuver, we tried to duplicate the real operative setting as closely as possible. The knee was held in 90° flexed position by one assistant and the maneuver was performed by the surgeon (*) while the other assistant inserted the pin through the guide. Additional maneuvers were applied to the transtibial technique while inserting the pin; 1) conventional transtibial technique (TT), 2) applying an anterior drawer force to the proximal tibia (TTA), 3) applying an anterior drawer force and a varus force to the proximal tibia (TTB) and 4) applying an anterior drawer force and a varus force to the proximal tibia and externally rotating the tibia and the femoral guide (mTT). After obtaining markings by using these four techniques, the tibial tunnel was re-reamed along the guide pin which was inserted to the position where the mTT was used with the maneuver. The diameter of the reamer was 10mm. Then, the medial femoral condyle was cut so as to evaluate the positions of markings on the medial wall of the lateral femoral condyle using the quadrant method (Fig 1) [12, 28]. The markings were not confirmed by arthroscope nor visually at each step, but it was evaluated after all the makings were made. The centers of the tibial tunnel articular apertures were also evaluated by the quadrant method (Fig 2) and the lengths of the short and long axes of the tibial tunnel apertures were measured (Fig 3). Digital photographs of each specimen were taken and uploaded to a personal computer. ImageJ software (National Institutes of Health, Bethesda, Maryland [http://rsb.info,nih.gov/ij/]) was used for measurements. This study was exempted from IRB review in accordance with the exemption criteria laid down by the institutional review board of our hospital. Cadavers were obtained from the hospital donation center, Catholic Institute For Applied Anatomy. None of the authors had direct contact with the study subjects.

### Statistical analysis

Comparisons of the femoral pin position of each step with the pin position of previous step were performed using a Wilcoxon signed-rank test after confirmation that the data were non-normally distributed data by Kolmogorov-Smirnov test. A p-value of <0.05 was considered significant. Two authors measured the parameters twice. The intraobserver and interobserver reliabilities in measurements were analysed using the intraclass correlation coefficient (ICC) (two-way random consistency).

## Results

**Fig 4:**
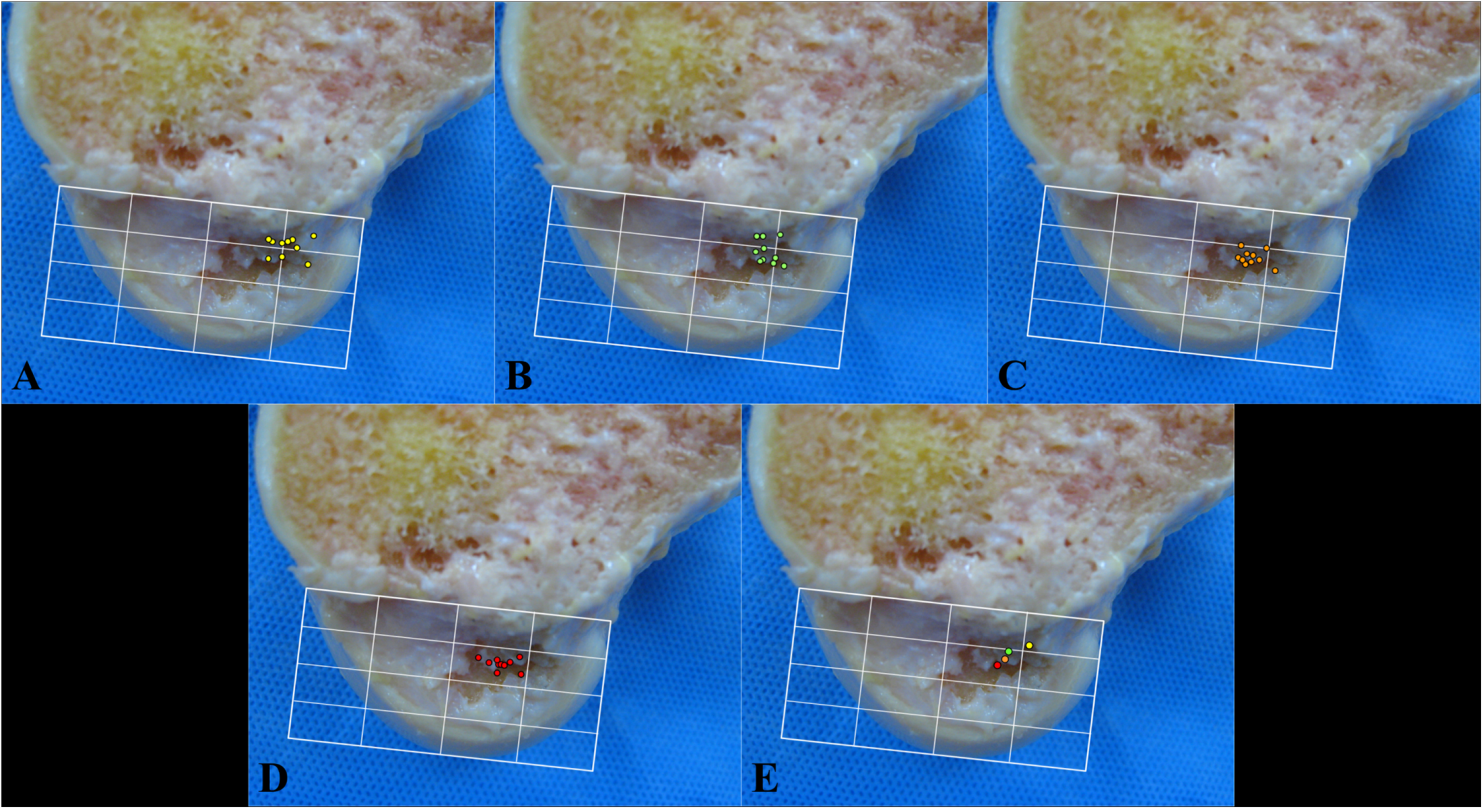
Femoral guide pin positions using four techniques in 10 fresh-frozen cadaveric knees. (A) Conventional transtibial technique (TT); (B) Applying an anterior drawer force to the proximal tibia (TTA); (C) Applying an anterior drawer force and a varus force to the promixal tibia (TTB); (D) Applying an anterior drawer force and a varus force to the proximal tibia and externally rotating the femoral guide pin and the tibia (mTT); (E) Average femoral guide pin points of each technique; Yellow circle, TT; Green circle, TTA; Orange circle, TTB; Red circle, mTT.

The femoral guide pin position shifted to more inferior and posterior position with each step of the mTT (all p values comparing the superior-inferior and anterior-posterior positions of each step with the positions of the previous step were 0.008 or less). The first step of applying an anterior drawer force resulted in the movement of the pin mainly to a more inferior position. The second step of applying an additional varus force resulted in the movement of the pin mainly to a more posterior position. The third step of external rotation of the femoral guide and the tibia resulted in the movement of the pin to a more inferior and posterior position (Fig 4, Table 1). The center of the tibial tunnel was placed at 42.0±3.7% in the anterior-posterior position and 50.9±1.9% in the medial-lateral position. The shape of the tibial tunnel aperture was oval without excessive tunnel expansion. It was 10.5±0.3mm wide and 12.9±1.1mm long.

**Table 1:** Femoral tunnel position evaluation using the quadrant method.

Quadrant method (%)		TT	TTA	TTB	mTT
Femur	Sup.-Inf.	23.6±4.5	28.4±3.4	30.1±3.8	33.2±4.5
	Ant.-Post.	23.9±4.3	26.2±3.7	32.0±4.3	36.9±4.8

The values are given as the mean and the standard deviation

Comparison of the pin position of each step with the pin position of previous step (Wilcoxon signed-rank test)

P-value of superior-inferior position comparison: Comparing TT group and TTA gourp– 0.005; TTA group and TTB group– 0.005; TTB group and mTT group– 0.005

P-value of anterior-posterior position comparison: Comparing TT group and TTA gourp– 0.008; TTA group and TTB group– 0.005; TTB group and mTT group– 0.005

Abbreviations: TT, conventional transtibial technique; TTA, applying an anterior drawer force to the proximal tibia; TTB, applying an anterior drawer force and a varus force to the proximal tibia; mTT, applying an anterior drawer force and a varus force to the proximal tibia and externally rotating the tibia and the femoral guide (modified transtibial technique); Sup., superior; Inf., inferior; Ant., anterior; Post., posterior

The ICC of all measurements was more than 0.8 which confirmed intraobserver and interobserver reliabilities of the measurements.

## Discussion

Conventional transtibial technique was first introduced to accomplish isometric graft placement and to avoid graft impingement [10]. Therefore, the tibial tunnel was created at a rather posterior position and the femoral tunnel was created at an anterior position which led to vertical graft placement [10, 11]. However, recent clinical and biomechanical studies demonstrated that anatomic positioning of the tunnels leads to better knee stability and graft function compared to that with isometric, vertical positioning of the tunnels [1, 2, 4–7, 29, 30]. Unfortunately, numerous studies have demonstrated that it is hard to achieve anatomical positioning of the tunnels using the transtibial technique [2, 7, 8, 11, 29, 31, 32]. Several authors have tried to modify the technique. Most of them concentrated on creating an oblique tibial tunnel for oblique placement of the graft [22–25]. Because the femoral tunnel is placed at a superior and anterior position and the tibial tunnel is placed at a posterior position using the transtibial technique, the modified technique should aim at moving the femoral tunnel to a more inferior and posterior position and the tibial tunnel to an anterior position. In order to move the femoral tunnel to the anatomic position we modified the transtibial technique by applying the three above mentioned steps. This study demonstrated that inferior and posterior movement of the femoral tunnel placement, adequate enough to be considered as an anatomic position of the ACL footprint, could be achieved using the mTT. The femoral guide pin position moved to more inferior and posterior with each step of the mTT.

In order to discuss whether the tunnels are placed in the anatomic positions during ACL reconstruction, identifying the anatomic positions of ACL footprints to a certain extent, if not accurate, should be preceded. There have been several cadaveric studies that investigated the position of ACL footprints. Although there were some differences among the data reported, we compared our data with these reports [12–21]. Small sample size and racial differences etc. might have played a role in yielding different results among the reports (Tables 2 and 3). Comparison of our study results with the previous cadaver studies showed that, the femoral tunnel position of 23.6±4.5% in the superior-inferior position and 23.9±4.3% in the anterior-posterior position by conventional transtibial technique resulted in a superior and anterior position as expected. After performing three steps of modification, the femoral tunnel was placed at 33.2±4.5% in the superior-inferior position and 36.9±4.8% in the anterior-posterior position, which is inferior and posterior enough to be considered as the anatomic position of ACL footprint [13–21] (Table 2). The tibial tunnel is created independently by transtibial technique, hence it is easier to control tibial tunnel placement. In our mTT, the tibial tunnel articular exit point was aimed at the center of ACL anteromedial and posterolateral bundle footprints. The tunnel was placed at 42.0±3.7% in the anterior-posterior position and 50.9±1.9% in the medial-lateral position, which also could be considered as the anatomic position based on previous cadaver studies [13, 14, 16–19, 21] (Table 3).

**Table 2:** Comparison of cadaver study results regarding the ACL femoral footprint centers using the quadrant method reported in the literature and this study.

Study		n	Superior-Inferior position (%)	Anterior-Posterior position (%)
Bernard et al.[12]		10	24.8	28.5
Musahl et al.[16]		8	26.6	26.3
Colombet et al.*[13]		7	29.4	36.5
Iriuchishima et al.*[20]		15	23.5	39.0
Takahashi et al.*[17]		32	35.9	40.1
Zantop et al.*[19]		20	23.9	38.0
Tsukada et al.*[18]		36	30.4	30.0
Pietrini et al.*[21]		12	25.3	28.3
Guo et al.[15]		16	43.1	38.3
Forsythe et al.*[14]		8	28.4	44.3
This study	TT	10	23.6	23.9
	TTA		28.4	26.2
	TTB		30.1	32.0
	mTT		33.2	36.9

*: Data were reported separately for anteromedial and posterolateral bundles in the original paper. Mean values of the two bundle data were presented in this table.

Abbreviations: n, number of cadaveric knees studied; AMB, anteromedial bundle; PLB, posterolateral bundle; TT, conventional transtibial technique; TTA, applying an anterior drawer force to the proximal tibia; TTB, applying an anterior drawer force and a varus force to the proximal tibia; mTT, applying an anterior drawer force and a varus force to the proximal tibia and externally rotating the tibia and the femoral guide (modified transtibial technique)

**Table 3:** Comparison of cadaver study results of the ACL tibial footprint centers using the quadrant method reported in the literature and this study.

Study	n	Anterior-Posterior	Medial-Lateral
Musahl et al.[16]	8	45.4	
Colombet et al.*[13]	7	44.0	
Takahashi et al.*[17]	31	30.4	48.3
Zantop et al.*[19]	20	37.0	
Tsukada et al.*[18]	36	43.9	48.9
Pietrini et al.*[21]	12	44.8	49.6
Forsythe et al.*[14]	8	35.7	51.5
This study	10	42.0	50.9

*: Data were reported separately for anteromedial and posterolateral bundles in the original paper. Mean values of the two bundle data were presented in this table.

Abbreviations: n, number of cadaveric knees studied; AMB, anteromedial bundle; PLB, posterolateral bundle; TT, conventional transtibial technique

There are concerns regarding expansion of the intra-articular tibial tunnel aperture with the use of the transtibial technique. Bedi et al. reported that considerable enlargement of the tibial tunnel aperture occurred due to iatrogenic re-reaming of the tibial tunnel along the eccentrically placed femoral guide wire used for placing the femoral tunnel in an anatomic position [2]. However in this study, the average lengths of the long and short axes of the tibial tunnel aperture were 12.9±1.1mm, 10.5±0.3mm respectively, and the tibial tunnel aperture was oval in shape but it was not excessively enlarged.

There were several limitations to this study. First, the maneuver study was performed on fresh-frozen cadaveric knees. There would be definite biomechanical differences between the fresh-frozen cadaveric knees and live human knees under anesthesia. However, the fresh-frozen cadaveric knee was the best available specimen that resembled the live human knee. Second, ages of the cadaver donors were much older than patients who usually receive ACL reconstruction. There could be substantial osteoarthritic changes presenting deformity and non-physiologic knee kinematics in these cadaveric knees. However, it was hard to obtain cadavers of young donors. Third, only ten cadaveric knees were used for this study. Although more number of subjects is always desirable in any anatomic study, there is a limited availability of cadavers. We think that the number of subjects in this study was not small compared to that in previous cadaver studies. Fourth, the maneuvers of the three stepwise techniques in our mTT were applied manually without any help of biomechanical instruments such as robotic or navigation system, which might give rise to inaccuracy and inconsistency of the study results. However, when we think of the real operative situation, these maneuvers are applied manually, not with the help of machines. Therefore, we think it is not inappropriate to conduct the study with manual maneuvers. Fifth, we did not use arthroscope to confirm the pin position on the femur while inserting the pin with each technique. Although, a medial parapatellar arthrotomy was used to excise ACL, the pin was inserted rather blindly with the patella reduced and the positions were checked with the patella everted to lateral side. In spite of the poor view, we could avoid extreme positionings. Finally, the measurements were made only by direct inspection or on the images taken with a digital camera. We tried to take radiographs or computed tomographic images of the specimen, however we were not permitted to do so.

## Conclusion

Single bundle ACL reconstruction and placement of the tunnels in the anatomic position without excessive tibial tunnel expansion can be achieved using the mTT by applying an anterior drawer force and a varus force to the proximal tibia and externally rotating the tibia and the femoral guide.

## Supporting information

S1 Table Femoral tunnel position data using the quadrant method of all specimen. (DOCX)

S2 Table Tibial tunnel position data using the quadrant method and the lengths of the short and long axes of the tibial tunnel apertures using mTT (all specimen). (DOCX)
