# Supplementary material for: Anatomic tunnel placement can be achieved with a modification to transtibial technique in single bundle anterior cruciate ligament reconstruction: A cadaver study
Source: PLoS One. 2017 Jul 31;12(7):e0180860. doi: 10.1371/journal.pone.0180860 (PMC5536285; doi:10.1371/journal.pone.0180860)
Supplement: S1 Table — (DOCX) [file pone.0180860.s001.docx]

S1 Table. Femoral tunnel position data using the quadrant method of all specimen

| Specimen | TT | | TTA | | TTB | | mTT | |
| --- | --- | --- | --- | --- | --- | --- | --- | --- |
|  | Sup.-Inf. | Ant.-Post. | Sup.-Inf. | Ant.-Post. | Sup.-Inf. | Ant.-Post. | Sup.-Inf. | Ant.-Post. |
| 1 | 25.33 | 22.22 | 29.33 | 22.22 | 30.67 | 30.56 | 33.33 | 38.89 |
| 2 | 23.68 | 20.51 | 28.95 | 25.64 | 31.58 | 35.90 | 34.21 | 43.59 |
| 3 | 25.00 | 27.27 | 29.76 | 29.55 | 32.14 | 34.09 | 35.71 | 38.64 |
| 4 | 26.19 | 22.22 | 32.14 | 26.67 | 34.52 | 31.11 | 36.90 | 37.78 |
| 5 | 24.44 | 21.74 | 31.11 | 26.09 | 33.33 | 32.61 | 35.56 | 34.78 |
| 6 | 15.31 | 18.18 | 24.49 | 20.45 | 25.51 | 25.00 | 27.55 | 27.27 |
| 7 | 28.75 | 21.52 | 31.91 | 22.22 | 33.29 | 25.95 | 40.28 | 32.42 |
| 8 | 22.25 | 24.32 | 24.89 | 27.50 | 26.44 | 32.22 | 28.89 | 35.14 |
| 9 | 28.23 | 29.11 | 29.79 | 29.17 | 30.77 | 33.30 | 33.94 | 37.33 |
| 10 | 16.52 | 32.05 | 22.11 | 32.12 | 23.04 | 39.30 | 26.04 | 42.70 |

The unit of the given values are percent

Abbreviations: TT, conventional transtibial technique; TTA, applying an anterior drawer force to the proximal tibia; TTB, applying an anterior drawer force and a varus force to the proximal tibia; mTT, applying an anterior drawer force and a varus force to the proximal tibia and externally rotating the tibia and the femoral guide (modified transtibial technique); Sup., superior; Inf., inferior; Ant., anterior; Post., posterior
