# Supplementary material for: Anatomic tunnel placement can be achieved with a modification to transtibial technique in single bundle anterior cruciate ligament reconstruction: A cadaver study
Source: PLoS One. 2017 Jul 31;12(7):e0180860. doi: 10.1371/journal.pone.0180860 (PMC5536285; doi:10.1371/journal.pone.0180860)
Supplement: S2 Table — (DOCX) [file pone.0180860.s002.docx]

S2 Table. Tibial tunnel position data using the quadrant method and the lengths of the short and long axes of the tibial tunnel apertures using mTT (all specimen)

| Specimen | Tibial Tunnel position | | Lengths of short and long axes of tibial tunnel | |
| --- | --- | --- | --- | --- |
|  | Ant.-Post. | Med.-Lat. | Long | Short |
| 1 | 47.6 | 50.0 | 11.5 | 10.2 |
| 2 | 39.5 | 51.4 | 11.5 | 11.0 |
| 3 | 38.5 | 53.4 | 15.0 | 10.4 |
| 4 | 42.6 | 52.8 | 13.5 | 10.3 |
| 5 | 37.8 | 52.1 | 13.0 | 10.6 |
| 6 | 45.2 | 48.9 | 12.0 | 10.3 |
| 7 | 43.9 | 52.0 | 13.5 | 10.7 |
| 8 | 46.1 | 50.8 | 12.5 | 10.4 |
| 9 | 37.0 | 47.2 | 12.8 | 10.9 |
| 10 | 42.2 | 50.8 | 13.3 | 10.2 |

The unit of tibial tunnel position values are percent and the unit of tibial tunnel axes length are millimeters.

Abbreviations: Ant., anterior; Post., posterior; Med., medial; Lat., lateral; mTT, modified transtibial technique
